# The Red Seaweed *Gracilaria gracilis* as a Multi Products Source

**DOI:** 10.3390/md11103754

**Published:** 2013-09-30

**Authors:** Matteo Francavilla, Massimo Franchi, Massimo Monteleone, Carmela Caroppo

**Affiliations:** 1 STAR-Agroenergy Research Group, University of Foggia, via Gramsci 89/91, Foggia 71121, Italy; E-Mail: massimo.monteleone@unifg.it; 2 Institute of Marine Science, National Research Council, via Pola 4, Lesina 71010, Italy; E-Mail: massimo.franchi@fg.ismar.cnr.it; 3 Institute for Coastal Marine Environment, National Research Council, via Roma 3, Taranto 74121, Italy; E-Mail: carmela.caroppo@iamc.cnr.it

**Keywords:** seaweed, biochemical composition, biorefinery, *Gracilaria*, Lesina Lagoon

## Abstract

In recent years seaweeds have increasingly attracted interest in the search for new drugs and have been shown to be a primary source of bioactive natural compounds and biomaterials. In the present investigation, the biochemical composition of the red seaweed *Gracilaria gracilis*, collected seasonally in the Lesina Lagoon (Southern Adriatic Sea, Lesina, Italy), was assayed by means of advanced analytical techniques, such as gas-chromatography coupled with mass spectrometry and spectrophotometric tests. In particular, analysis of lipids, fatty acids, sterols, proteins, phycobiliproteins and carbohydrates as well as phenolic content, antioxidant and radical scavenging activity were performed. In winter extracts of *G. gracilis*, a high content of R-phycoerythrin together with other valuable products such as arachidonic acid (PUFA ω-6), proteins and carbohydrates was observed. High antioxidant and radical scavenging activities were also detected in summer extracts of the seaweed together with a high content of total phenols. In conclusion, this study points out the possibility of using *Gracilaria gracilis* as a *multi products source* for biotechnological, nutraceutical and pharmaceutical applications even although more investigations are required for separating, purifying and characterizing these bioactive compounds.

## 1. Introduction

The marine world, due to its phenomenal biodiversity, is a rich natural resource of many biologically active compounds [1,2]. Many marine organisms live in complex habitats exposed to extreme conditions and, in adapting to new environmental surroundings, they produce a wide variety of primary and secondary metabolites which cannot be found in other organisms. Marine-based bioactive compounds can be derived from a vast array of sources, including marine plants, macro- and microalgae, microorganisms, and sponges, all of which contain their own unique set of biomolecules [3].

Macroalgae, known also as seaweeds, produce many biologically active phytochemicals, which include among others, carotenoids, terpenoids, xanthophylls, chlorophylls, phycobilins, polyunsaturated fatty acids, polysaccharides, vitamins, sterols, tocopherol and phycocyanins [4]. Seaweeds represent 23.4% of the tonnage and 9.7% of the value of the global (marine, brackish water, and freshwater) aquaculture production, estimated at 59.4 million tonnes and $ 70.3 billion in 2004 [5,6]. They are used as food, fodder, feed and fertilizer [7] and many of the bioactive compounds produced by the macroalgae are known to have potential beneficial use in healthcare [8,9].

*Gracilaria* Greville genus (Gracilariales, Rhodophyta) is represented by more than 300 species of which 160 have been accepted taxonomically. The macroalgae belonging to this genus are important for industrial and biotechnological uses and are considered economically valuable resources, because of their ability to achieve high yields of commercially valuable biomass [10]. In fact, they contain, besides other compounds, phycocolloids, the main source of agar-agar, which is a gelatinous non-toxic colloidal carbohydrate present in the cell wall and intercellular spaces of the algae and has wide use in the preparation of food, ice creams, jellies, soups, bacteriological samples and cosmetics [7,11]. These algae are also sources of important bioactive metabolites with antibiotic activity; but also sources of different prostaglandins and other substances that may be toxic to humans by causing gastrointestinal disorders and lethality [4].

During the period 1970–1990, in the Mediterranean Lesina Lagoon (Southern Adriatic Sea, Lesina, Foggia, Italy) local fishermen used to harvest the red seaweed *Gracilaria gracilis* (Stackhouse) Steentoft, Irvine *et* Farnham (as *Gracilaria confervoides* Greville) [12,13]. The biomass, after the sun drying process, was sold to some private companies as raw material for agar extraction. The harvested algal biomass reached 100 t dried weight per year [14]. Unfortunately, the over-exploitation of this natural resource contributed to a drastic reduction in production of *Gracilaria* biomass. As a consequence, other seaweeds not economically valuable, like *Valonia aegagropila*, *Chaetomorpha* sp*.* grew quickly in the Lesina Lagoon, reaching sometimes bloom conditions [15,16].

After this period, some field experiments aimed at re-establishing stable coverage of *Gracilaria gracilis* were carried out successfully in the Lesina Lagoon. These studies pointed out not only an increase in *Gracilaria* biomass (around 1200 t wet weight per year), but also the nutrient uptake capacity of this species in the area of its re-colonization in the lagoon [17]. This aspect is particularly interesting taking into account that *Gracilaria* could also represent a bio-remediator useful to control the risk of eutrophication in this coastal lagoon.

Recently, Francavilla *et al*. [18] found that Gracilaria harvested in the Lesina Lagoon could be used as an interesting source of natural porous material with several biotechnological applications. Moreover, Buldarin *et al*. [19] found that microwave (MW)-mediated pyrolysis of this macroalga produced chemical rich bio-oils which are rich in aromatics, sugars and other high value chemicals.

In the light of those results and of worldwide great interest in algae biomass as a source of bioactive compounds (for the nutraceutical, pharmaceutical and cosmetic industry) and new biomaterials, the aim of this work was to screen (for the first time, to the best of our knowledge) the biochemical composition of *Gracilaria gracilis* collected in the Lesina Lagoon, with the idea of taking into account the algal biomass as a hypothetical *multi product source*. In particular, the analysis of lipids, fatty acids, sterols, protein, phycobiliproteins, carbohydrates, as well as phenolic content, antioxidant and radical scavenging activity were performed. Moreover, because biochemical composition depends on many environmental and seasonal factors [20,21], *Gracilaria* was collected seasonally in order to evaluate if there were significant differences in the biochemical composition of this species.

## 2. Results and Discussion

### 2.1. Lipids and Fatty Acids (FAMEs)

Lipids are a large group of natural compounds which includes fats, waxes, sterols, fat-soluble vitamins (such as vitamins A, D, E and K), monoglycerides, diglycerides, phospholipids, carotenoids and others [22]. They play many biological functions including energy storage, structural components of cell membranes, and signalling molecules. Although humans and other mammals use various biosynthetic pathways to both break down and synthesize lipids, some essential lipids can be obtained only from diet [22].

**Figure 1:**
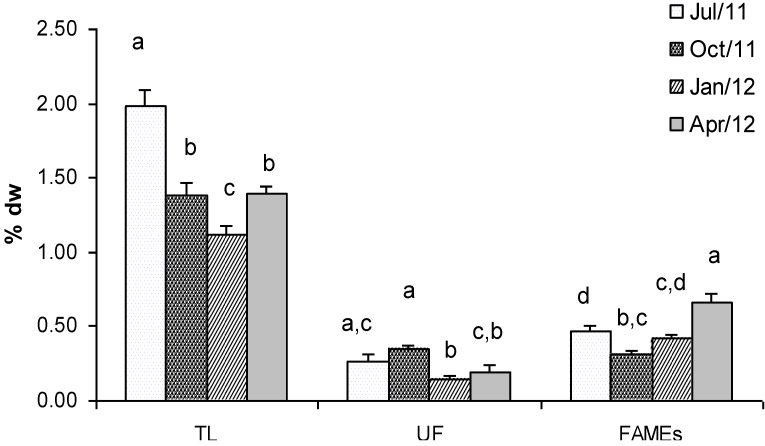
Mean concentration (% dry weight) of Total Lipids (TL), Unsaponified fraction (UF) and Fatty Acids Methyl Esters (FAMEs) extracted from *G. gracilis.* Different superscripts (a–d) indicate significant difference (*p* < 0.05).

Figure 1 shows the concentration of total lipids (TL), unsaponified fraction (UF) and fatty acids (FAMEs) extracted from *Gracilaria* seaweed. The biomass sampled in July showed the highest lipid content which represented 1.98% dry weight The lowest concentration in TL was found in winter (January, 1.12% dry weight), whereas intermediate concentrations were found in *Gracilaria* sampled in October and April (1.38% and 1.40% dry weight, respectively). Statistical analyses confirmed that TL values were significantly different in all the seasons, only with comparable values in fall and spring (Figure 1). The detected concentrations were very similar to those measured by other authors in *Gracilaria* sp*.* [23,24] and confirmed the low lipid content in red seaweeds. Therefore, its contribution as a food energy source appears to be low.

The unsaponified fraction of total lipids, which includes waxes, sterols, fat-soluble vitamins and carotenoids, generally was low and ranged between 0.35% dry weight in October and 0.15% dry weight in January (Figure 1). In this case Tukey’s test showed significant differences only in the samples collected in autumn and winter (Figure 1).

With regard to the total amount of FAMEs in *Gracilaria*, the highest concentration was observed in biomass collected in April (0.67% dry weight) whereas the lowest concentration was in October (0.31% dry weight). Statistical analyses showed significant differences in terms of FAMEs content between samples collected in April and those sampled in the other months (Figure 1). No significant differences were found between samples collected in summer and winter, while samples collected in winter were similar (*p* > 0.05) to those collected in autumn. Fatty acids are precursors in the biosynthesis of eicosanoids, which are important bioregulators of many cellular processes [25,26,27]. Eicosapentaenoic (EPA) and docosahexaenoic (DHA) polyunsaturated ω-3 fatty acids are known to: (i) have capacities for cardioprotection; (ii) reduce triacylglycerol and cholesterol levels; (iii) have antiinflamatory and anticancer effects [28]. FAMEs composition in *Gracilaria*, a mixture of 31 compounds, showed significant variation between the analysed seasons (Table 1).

**Table 1:** Fatty Acids composition (% w/w of total amount of fatty acids) of *G. gracilis* sampled in four different seasons.

Fatty Acids Methyl Esters	Structure	Fatty Acid Content (%)			
		11 July	11 October	12 January	12 April
Methyl decanoate	C10:0	0.05 ^a^	0.03 ^b^	nd	0.03 ^b^
Methyl undecanoate	C11:0	0.01	nd	nd	nd
Methyl dodecanoate	C12:0	0.28 ^a^	0.35 ^a^	0.06 ^b^	0.12 ^c^
Methyl tridecanoate	C13:0	0.06 ^a^	0.07 ^a^	0.02 ^b^	0.03 ^b^
Methyl 9-tetradecenoate	9-C14:1	0.02 ^a^	0.39 ^b^	0.74 ^c^	0.55 ^d^
Methyl tetradecanoate	C14:0	5.48 ^a^	5.55 ^a^	3.48 ^b^	5.13 ^a^
*cis*-10 Pentadecenoic Acid Methyl Ester	10-C15:1	nd	0.15 ^a^	0.03 ^b^	0.04 ^b^
Pentadecanoic Acid Methyl Ester	C15:0	1.03 ^a^	1.32 ^b^	0.52 ^c^	0.53 ^c^
Palmitoleic Acid Methyl Ester	9-C16:1	3.84 ^a^	8.19 ^b^	2.21 ^c^	8.81 ^d^
Palmitic Acid Methyl Ester	C16:0	25.67 ^a^	38.13 ^b^	28.55 ^c^	29.29 ^c^
*cis*-10 Heptadecenoic Acid Methyl Ester	10-C17:1	0.14 ^a^	1.12 ^b^	0.23 ^c^	0.24 ^c^
Heptadecanoic Acid Methyl Ester	C17:0	0.64 ^a^	1.14 ^b^	0.20 ^c^	0.30 ^c^
Linolenic Acid Methyl Ester	C18:3*n*-3	0.42 ^a^	0.23 ^b^	0.09 ^c^	3.88 ^d^
Linoleic Acid Methyl Ester	C18:2*n*-6c	4.85 ^a^	2.49 ^b^	1.33 ^c^	4.14 ^d^
Oleic Acid Methyl Ester	C18:1*n*-9c	10.78 ^a^	8.79 ^b^	5.76 ^c^	9.12 ^b^
Elaidic Acid Methyl Ester	C18:1*n*-9t	4.16 ^a^	6.15 ^b^	3.22 ^c^	2.02 ^d^
Stearic Acid Methyl Ester	C18:0	3.35 ^a^	3.62 ^a^	1.87 ^b^	2.30 ^c^
Arachidonic Acid Methyl Ester	C20:4*n*-6	33.27 ^a^	16.05 ^b^	47.78 ^c^	38.30 ^d^
*cis*-5-8-11-14-17-Eicosapentaenoic Acid Methyl Ester	C20:5*n*-3	1.13 ^a^	1.84 ^b^	0.24 ^c^	3.93 ^d^
*cis*-11,14,17-Eicosatrienoic Acid Methyl Ester	C20:3*n*-3	2.48 ^a^	1.23 ^b^	2.07 ^c^	2.82 ^a^
*cis*-11,14-Eicosdienoic Acid Methyl Ester	11,14-C20:2	0.32 ^a^	0.25 ^b^	0.23 ^b,c^	0.19 ^c^
*cis*-8,11,14-Eicosatrienoic Acid Methyl Ester	8,11,14-C20:3*n*-6	nd	0.32 ^a^	0.71 ^b^	nd
*cis*-11 Eicosenoic Acid Methyl Ester	11-C20:1	0.28	nd	nd	nd
Arachidic Acid Methyl Ester	C20:0	0.15 ^a^	0.40 ^b^	0.08 ^c^	0.61 ^d^
Heneicosanoic Acid Methyl Ester	C21:1	0.02 ^a^	0.02 ^a^	0.04 ^b^	0.02 ^a^
*cis*-4,7,10,13,16,19-Docosahexaenoic Acid Methyl Ester	C22:6*n*-3	0.23 ^a^	0.43 ^b^	0.03 ^c^	0.12 ^d^
Erucidic Acid Methyl Ester	C22:1*n*-9	0.65 ^a^	0.34 ^b^	0.19 ^c^	0.13 ^c^
Docosanoic Acid Methyl Ester	C22:0	0.26 ^a^	0.38 ^b^	0.09 ^c^	0.19 ^d^
Tricosanoic Acid Methyl Ester	C23:0	0.08 ^a^	0.16 ^b^	0.02 ^c^	0.02 ^c^
*cis*-15-Tetracosenoic Acid Methyl Ester	15-C24:1	0.09 ^a^	0.31 ^b^	0.15 ^c^	0.15 ^c^
Tetracosanoic Acid Methyl Ester	C24:0	0.27 ^a^	0.56 ^b^	0.05 ^c^	0.15 ^d^
TOTAL (mg g^−1^ dry weight)		4.71 ^a^	3.14 ^b^	4.18 ^a^	6.67 ^c^
SFA (% w/w)		37.33 ^a^	51.71 ^b^	34.94 ^c^	34.19 ^c^
MUFA (% w/w)		19.98 ^a^	25.31 ^b^	12.54 ^c^	18.60 ^a^
PUFA (% w/w)		42.70 ^a^	22.52 ^b^	51.77 ^c^	47.18 ^a^
PUFAω6 (% w/w)		38.12 ^a^	18.86 ^b^	49.82 ^c^	37.51 ^a^
PUFAω3 (% w/w)		4.26 ^a^	3.73 ^a,b^	2.43 ^b^	9.51 ^c^
ω6/ω3		8.96 ^a^	5.06 ^b^	20.48 ^c^	3.95 ^b^

SFA: Saturated Fatty Acids; MUFA: Mono-Unsaturated Fatty Acids; PUFA: Poly-Unsaturated Fatty Acids. ω6/ω3: ratio of ω6 and ω3 Fatty Acids; nd: not detected. ^a–d^ Row wise values with different superscripts of this type indicate significant difference (*p* < 0.05).

In July, the most abundant FAMEs were arachidonic acid (AA-C20:4 *n*-6) and palmitic acid (PA-C16:0). AA reached the highest relative concentration during winter time (January, 48%). The lowest content in AA was found in October (16%) whereas intermediate concentrations were observed in July and April (33% and 38% respectively). Tukey’s test revealed significant differences in AA concentration in samples collected in all the seasons (Table 1). Concerning PA, its highest concentration was detected in October (38%) when AA was less concentrated. The lowest value was observed in July (26%). Statistical analyses confirmed significant differences in terms of PA concentration only in these two periods (Table 1). Other abundant fatty acids monitored in *Gracilaria* were: tetradecanoic acid (C14:0), palmitoleic acid (9-C16:1), stearic acid (C18:0), oleic acid (C18:1*n*-9c), elaidic acid (C18:1*n*-9t) and linoleic acid (C18:2*n*-6c). PUFAs (Poly-Unsaturated Fatty Acids) were 52% by total FAMEs in January, when also the lowest content in MUFAs (Mono Unsaturated Fatty Acids) and SFAs (Saturated Fatty Acids) was found (20% and 37%, respectively) (Table 1). In October, concentration of PUFAs decreased to 23% and concentration of MUFAs and SFAs reached their highest values (25% and 52%, respectively). In April, when the highest content in FAMEs was found (0.67% dry weight), PUFAs were 47% of total FAMEs and MUFAs and SFAs represented 19% and 34% respectively. Arachidonic acid and linoleic acid were the only two PUFAs ω-6 present in *Gracilaria*, whereas linolenic acid (C18:3*n*-3), eicosapentanoic acid (C20:5*n*-3), eicosatrienoic acid (C20:3*n*-3) and docosahexanoic acid (C22:6*n*-3) were the PUFAs ω-3 detected.

Statistical analysis showed that in *Gracilaria* biomass the concentrations of palmitoleic acid, elaidic acid, linoleic acid, eicosapentanoic acid and docosahexanoic acid were significantly different in all the seasons. Also the other fatty acids showed significant differences in their concentration but in a reduced number of seasons (Table 1).

Recently, the importance of the ω-6/ω-3 ratio has been discussed in scientific reports. The original value 1 of the ratio ω-6/ω-3 involved the balance of intake of both polyunsaturated ω-6 and ω-3 fatty acids. The high dietary intake of ω-6 fatty acids from a diet rich in vegetable oils causes detrimental turnover of the balance ratio (ω-6/ω-3) to ω-3 fatty acids. This ratio is 50 in Europe and the United States, and 12 in Japan, which is compared to 1 for Greenland Eskimos due to their higher consumption of fish fatty acids [29]. A huge intake of ω-6 fatty acids and an excessively high ω-6/ω-3 ratio promotes the pathogenesis of cardiovascular, inflammatory, and autoimmune diseases and cancer [30]. The WHO currently recommends a ω-6/ω-3 ratio lower than 10. The ω-6/ω-3 ratio in *Gracilaria* extracts was constantly below this limit. The lowest value was found in samples collected in April when the highest amount of FAMEs was found too. Only in January was this ratio higher than 10 because of the high concentration of AA (48%, Table 1). Therefore, taking into account all the results in terms of FAMEs, content of PUFAs and ω-6/ω-3 ratio, *Gracilaria* biomass seems to be an interesting candidate as source of PUFAs and AA if it is harvested in spring, according also to the results reported by MacArtain *et al*. [31].

### 2.2. Sterols

The composition of the sterols fraction extracted from *Gracilaria* was investigated in order to explore if phytosterols, sterols with C_28_ and C_29_ structure, were present. This is because of the great interest in these compounds for their bioactivity and promising nutraceutical and pharmaceutical use. In fact, they are precursors of some bioactive molecules (e.g., ergosterol is a precursor of Vitamin D2) and have also been shown to lower total and LDL cholesterol levels in humans by inhibiting cholesterol absorption from the intestine [32]. Moreover, phytosterols possess anti-inflammatory activity and may be characterized by anti-cancer and anti-oxidative activities [33].

**Figure 2:**
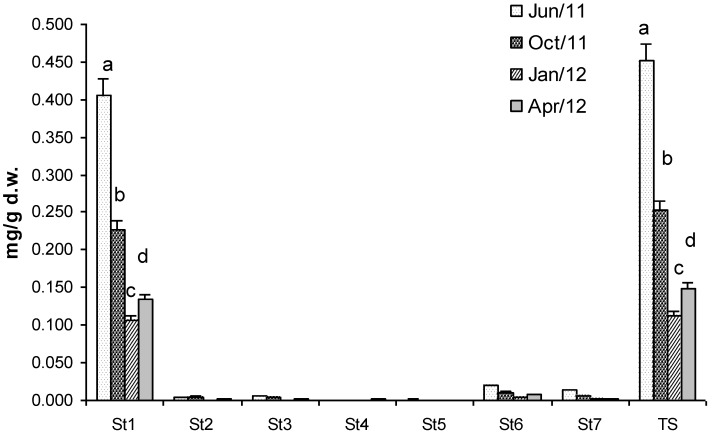
Concentration (mg g^−1^ dry weight of biomass) of Total Sterol (TS) and sterols identified in *G. gracilis*. Cholesterol (*St*_1_), (22*Z*)-ergosta-7,22-dien-3β-ol (*St*_2_), (24*R*)-methylcholest-5-en-3β-ol (*St*_3_), ergostan-5α-3β-ol (*St*_4_), (24*S*) stigmasta 5,22-dien-3β-ol (*St*_5_), Stigmast-5-en-3β-ol (*St*_6_) and stigmastan-5α-3β-ol (*St*_7_). Different superscripts (a–d) indicate significant difference (*p* < 0.05).

Total sterols (TS) concentration, analysed in the unsaponified fraction, was 0.45 mg/g dry weight of biomass in July (Figure 2). This value decreased drastically in January when TS was 0.11 mg/g dry weight cholesterol and TS concentrations were statistically different in all the samples of *Gracilaria* (Figure 2). Seven sterols were identified and corresponded respectively to: cholesterol (*St*_1_), (22*Z*)-ergosta-7,22-dien-3β-ol (*St*_2_), (24*R*)-methylcholest-5-en-3β-ol (*St*_3_), ergostan-5α-3β-ol (*St*_4_), (24*S*) stigmasta 5,22-dien-3β-ol (*St*_5_), Stigmast-5-en-3β-ol (*St*_6_) and stigmastan-5α-3β-ol (*St*_7_). Unfortunately, cholesterol was the most abundant, representing about 90% of TS. *St6* and *St7*, two phytosterols, were 4% and 3% of TS respectively, whereas the remaining sterols were only in traces. Therefore, unfortunately, *Gracilaria* biomass cannot be considered as a source of phytosterols for nutraceutical applications because of the high content of cholesterol.

### 2.3. Proteins

Macroalgae are a unique source of proteins although the content is very variable. Proteins are composed of different amino acids and hence the nutritional quality can be determined basically by the content, proportion and availability of these amino acids. Proteins can have antibacterial, antioxidant, immunostimulating, antithrombotic and anti-inflammatory activities; they can be used for prevention and treatment of hypertension, diabetes and hepatitis among other positive effects in the organism. All these health promoting effects make these compounds of great relevance as nutraceuticals [22]. Analyses of total protein in algae are often done in order to search for new sources of protein supplements. In *Gracilaria* species the protein content found ranged from 5.6% to 30% [34,35,36,37,38].

**Figure 3:**
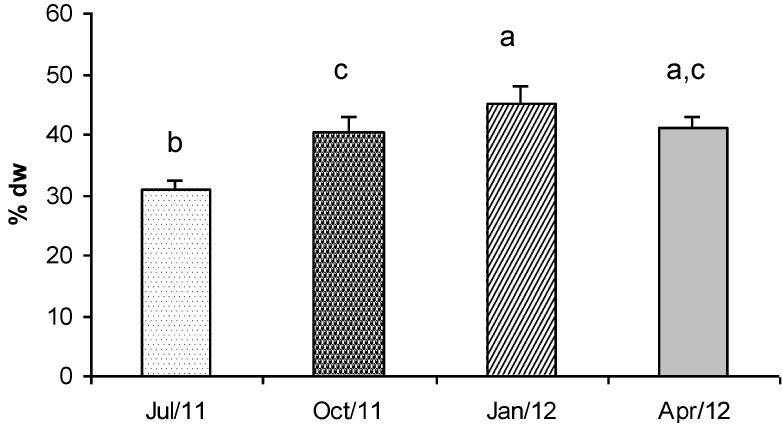
Concentration (% dry weight) of total proteins in *G. gracilis*. Different superscripts (a–d) indicate significant difference (*p* < 0.05).

In this study, *Gracilaria* showed a very interesting protein concentration (Figure 3). In particular the protein content observed in January was 45% dry weight This value decreased in October and April (about 41% dry weight) and reached the lowest concentration in summer (July, 31% dry weight).

Tukey’s test revealed that the protein concentrations were significantly different in all the seasons, except for the samples collected in April when the concentrations were significantly different only with respect to the value detected in July (Figure 3). Therefore, macroalgal biomass is a very promising source of protein if it is harvested in winter and spring.

### 2.4. Phycobiliproteins

The phycobiliproteins are proteins with linear tetrapyrrole prosthetic groups (bilins) that, in their functional state, are covalently linked to specific cysteine residues of the proteins. These proteins are found in cyanobacteria (blue-green algae), in a class of biflagellate unicellular eukaryotic algae (cryptomonads), and in Rhodophyta (red algae). In all of them the phycobiliproteins act as photosynthetic accessory pigments.

**Figure 4:**
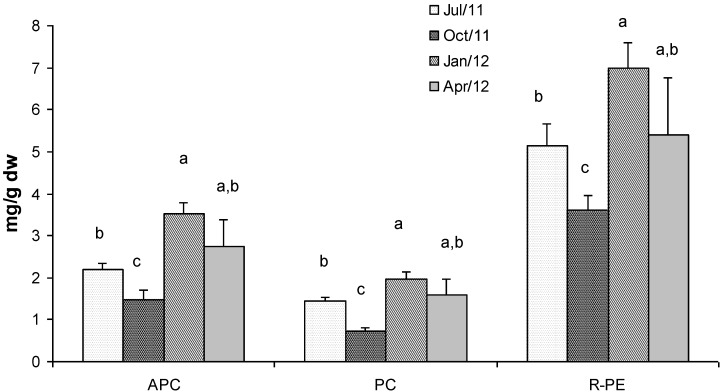
Concentration (mg g^−1^ dry weight) of phycobiliproteins in *G. gracilis*. Allophycocyanin (APC), phycocyanin (PC) and R-phycoerythrin (R-PE). Different superscripts (a–d) indicate significant difference (*p* < 0.05).

Figure 4 shows the concentrations of phycobiliproteins in *Gracilaria* extracts. R-phycoerythrin (R-PE) is the most abundant with a concentration which ranged between 7 mg/g dry weight in January and 3.6 mg/g dry weight in October. Allophycocyanin (APC) showed lower concentration than R-PE which ranged between 3.5 mg/g dry weight and 1.5 mg/g dry weight, in January and October respectively. Phycocyanin (PC) was the less abundant phycobiliprotein showing a concentration that ranged from 3 mg/g dry weight (d.w.) in January to 0.7 mg/g d.w. in October. Generally, the highest concentrations of the three phycobiliproteins were found in biomass sampled in winter time when light radiation and nutrient concentration in the lagoon are favourable for the biosynthesis of these metabolites [17].

Statistical analyses showed significant differences in terms of phycobiliprotein concentration in all the seasons, except for samples collected in April when the concentrations were significantly different only with respect to the values detected in October (Figure 4).

Phycobiliproteins can be used as very useful fluorescent probes because of their excellent spectroscopic properties [39,40], stability, high absorption coefficients, and high quantum yields. They are highly soluble in water and exhibit a large Stokes shift which is very important for detection [41]. Phycobiliproteins are attractive since they are not harmful to humans if they are applied to an external surface or ingested. They are already used as photosensitizers for treatment of tumors and have potential to substitute Photofrin (a kind of light sensitive agent in photodynamic therapy) in common use which is purified from animal blood [42]. Phycobiliproteins are also widely used as natural colorants for food and cosmetics. For their wide usage phycobiliproteins have a great economic potential [43,44]. The concentration of R-PE found in *Gracilaria* harvested in winter is very interesting from a biotechnological point of view. Therefore, the algal biomass could be proposed as a novel industrial source of R-PE. Actually, purified R-PE is a very expensive compound which costs about 50 euro per mg (AnaSpec, Inc., Fremont, CA, USA).

### 2.5. Fractionated Extraction of Algal Biomass for Antioxidant Assays and Analysis of Total Phenolic Content

**Figure 5:**
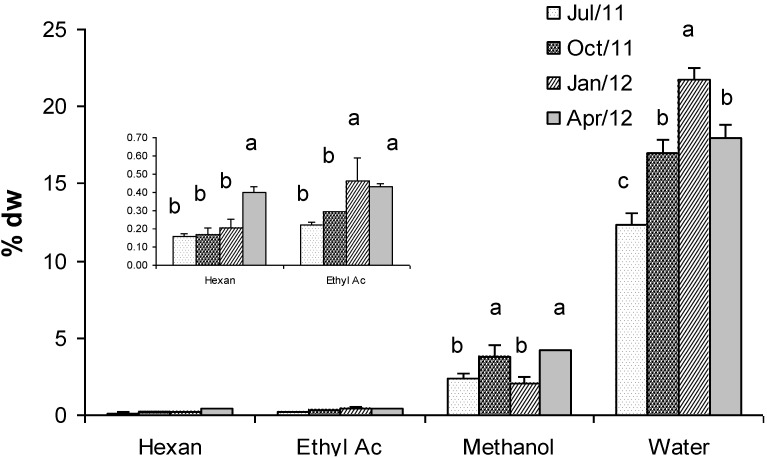
Concentration (% dry weight) of extracts of *G. gracilis* obtained by using four solvents with increasing polarity: *n*-hexane, ethyl acetate, methanol and deionized water (at 80 °C). Different superscripts (a–d) indicate significant difference (*p* < 0.05).

Figure 5 shows concentrations of *Gracilaria* extracts obtained by using four solvents with increasing polarity: *n*-hexane, ethyl acetate, methanol and water (at 80 °C). Results evidenced that the higher the solvent polarity the higher the concentration of extract. Therefore, the highest concentration was found using water (21% dry weight), whereas the lowest one was found using *n*-hexane (0.16% d.w.). Statistically significant differences were detected mainly in the water extracts during all the seasons, except in April when values were comparable with those observed in October (Figure 5). Methanol and ethyl acetate showed intermediate concentrations (4.2% and 0.43% dry weight in April respectively). Generally, extracts of biomass in winter were more concentrated than extracts of biomass in other seasons. Exceptions were represented by the *n*-hexane and methanol extracts that showed the highest concentration in April (0.40% dry weight). Interestingly, in the same sample the highest concentrations of FAMEs were also found, which might have been extracted by *n*-hexane.

The dried extracts, dissolved in methanol, were used for antioxidant assays and analysis of phenolic content.

### 2.6. Antioxidant Activity Assays

Reactive oxygen species such as hydroxyl, super oxide and peroxyl radicals produced in human tissue cells are responsible for extensive oxidative damage that leads to age related degenerative conditions, cancer and a wide range of other human diseases [45,46].

Many researchers have found different types of antioxidants in various species of higher plants [47,48]. However, seaweeds also are considered to be an interesting source of antioxidant compounds. In fact, several researchers have reported the antioxidant properties of both brown and red seaweeds from across the globe [49,50,51,52]. Further, the evidence available in the literature suggests the potential protective effects of seaweeds against oxidative stress in target tissues and lipid oxidation in food [53]. To date no reports have identified *Gracilaria gracilis* as a source of antioxidant compounds. While there are two reports of *Gracilaria edulis* showing antioxidant activity it is not possible to compare the antioxidant results reported here and those reported in *Gracilaria edulis* as different extractions methods were used [51,54].

Many authors have stressed the need to carry out more than one type of antioxidant activity measurement to take into account the various mechanisms of antioxidant action [52,55], as no single assay will accurately reflect all of the radical sources or all antioxidants in a mixed or complex system [56].

Three different antioxidant assays were carried out in this study to test the activity of *Gracilaria* extracts: FRAP, ABTS and DPPH assays.

#### 2.6.1. Ferric-Reducing Antioxidant Power (FRAP) Assay

The four seasonal extracts showed a high variability in antioxidant activity (as µmol Trolox g^−1^ extract) (Table 2). Analysing the biomass sampled in July, the highest activity (809 µmol Trolox g^−1^) was found in the ethyl acetate extract. The n-hexane extract showed an intermediate value (370 µmol Trolox g^−1^) whereas in the methanol and water fraction the lowest antioxidant activities were found (100 and 13 µmol Trolox g^−1^, respectively). Interestingly, these values decreased to about 60%–70% in extracts of biomass sampled in October. In fact, the antioxidant activity was 312 µmol Trolox g^−1^ in the ethyl acetate extract, 114 µmol Trolox g^−1^ in hexane, 27 µmol Trolox g^−1^ in methanol and 9 µmol Trolox g^−1^ in the water extract.

**Table 2:** Ferric-reducing antioxidant power (FRAP) (µmol Trolox g^−1^ extract) of extracts obtained from *G. gracilis* sampled in four different seasons (*n* = 3).

	Extracts				
Date	HE	EA	ME	WT	CTR (BHT)
July/11	370.74 ± 18.5 ^a^	808.90 ± 40.4 ^a^	99.90 ± 5.0 ^a^	13.32 ± 0.7 ^a^	1917.93 ± 95.9
October/11	114.28 ± 5.7 ^b^	312.47 ± 15.6 ^b^	26.90 ± 1.3 ^b^	9.06 ± 0.5 ^b^	
January/12	189.23 ± 9.5 ^c^	62.02 ± 3.1 ^c^	53.05 ± 2.7 ^c^	9.19 ± 0.5 ^b^	
April/12	117.24 ± 5.9 ^b^	29.82 ± 1.5 ^d^	50.14 ± 2.5 ^c^	14.61 ± 0.7 ^a^	

All the values are mean ± SD; SD: standard deviation.HE: n-Hexan; EA: Ethyl Acetate; ME: methanol; WT: Water (80 °C); CTR: Control (butylated hydroxytoluene). ^a–d^ Column wise values with different superscripts of this type indicate significant difference (*p* < 0.05).

Statistical analyses evidenced significant differences in all the seasons with regard to the ethyl acetate extracts. In the *n*-hexane and methanol extracts differences were evidenced in all the seasons except in April when values were comparable with those detected in October and January, respectively. In water extracts values showed a lower seasonality (Table 2).

Comparing these results with a known and commercial antioxidant, butylated hydroxytoluene (BHT), we found that the ethyl acetate extract (of July) had 42% antioxidant activity by BHT (1918 µmol Trolox g^−1^) (Table 2).

#### 2.6.2. ABTS Assay

Results of ABTS antioxidant assay are reported in Table 3 (as mmol Trolox g^−1^ extract). Similar to that found using FRAP assay, the highest activity was found in the ethyl acetate extract of biomass sampled in July (0.43 mmol Trolox g^−1^). However, this activity decreased only marginally in April (0.34 mmol Trolox g^−1^). The hexane extract showed the highest activity in April (0.26 mmol Trolox g^−1^) and the lowest in October (0.07 mmol Trolox g^−1^). Interestingly, in the methanol and water extracts of *Gracilaria* seaweed the lowest antioxidant activities were found. They ranged between 0.06 and 0.02 mmol Trolox g^−1^ for methanol extracts and between 0.15 and 0.05 mmol Trolox g^−1^ for water extracts. As already observed for the FRAP assay, Tukey’s test showed significant differences in all the seasons with regard to the ethyl acetate extracts. In the *n*-hexane extracts differences were evidenced in all the seasons except in October when values were comparable with those detected in July. In the methanol and water extracts values showed a lower seasonality (Table 3).

Surprisingly, antioxidant activity of ethyl acetate extracts for July and April were higher than the activity of gallic acid (0.28 mmol Trolox g^−1^) used as control.

#### 2.6.3. DPPH Assay

Table 4 shows the scavenging activity of extracts of *Gracilaria* biomass at different concentrations. EC_50_ value (mg extract mL^−1^), the effective concentration at which 50% of the DPPH radicals were scavenged, is used to compare the scavenging activity of extracts.

**Table 3:** Antioxidant activity (mmol Trolox g^−1^ extract) of extracts obtained from *G. gracilis* sampled in four different seasons measured using ABTS assay (*n* = 3).

	Extracts				
Date	HE	EA	ME	WT	CTR (GA)
July/11	0.09 ± 0.02 ^a^	0.43 ± 0.04 ^a^	0.06 ± 0.01 ^a^	0.07 ± 0.02 ^a^	0.28 ± 0.04
October/11	0.07 ± 0.01 ^a^	0.18 ± 0.02 ^b^	0.02 ± 0.01 ^b^	0.05 ± 0.01 ^a^	
January/12	0.16 ± 0.03 ^b^	0.26 ± 0.02 ^c^	0.06 ± 0.02 ^a^	0.10 ± 0.03 ^a,b^	
April/12	0.26 ± 0.03 ^c^	0.34 ± 0.03 ^d^	0.03 ± 0.01 ^b^	0.15 ± 0.03 ^b^	

All the values are mean ± SD; SD: standard deviation; HE: *n*-Hexan; EA: Ethyl Acetate; ME: methanol; WT: Water (80 °C); CTR: Control (gallic acid). ^a–d^ Column wise values with different superscripts of this type indicate significant difference (*p* < 0.05).

The ethyl acetate extract of seaweed sampled in July showed the highest scavenging effect, corresponding to the lowest EC_50_ (0.82 mg mL^−1^) (Table 4). The hexane extract of the same sample of biomass (July), showed intermediate EC_50_ value (1.1 mg mL^−1^) and methanol showed the highest value (2.94 mg mL^−1^). At the concentrations tested, it was not possible to measure the Ec_50_ of the water extract. Therefore, we reported EC_40_, the concentration at which 40% of the DPPH radicals were scavenged. The EC_40_ of the water extract of July was 10 mg mL^−1^.

**Table 4:** DPPH radical scavenging activity (EC_50_ value, mg extract mL^−1^ methanol) in extracts of *G. gracilis* sampled in four different seasons (*n* = 3).

	Extracts				
Date	HE	EA	ME	WT	CTR (BHT)
11 July	1.1 ± 0.06 ^a^	0.82 ± 0.04 ^a^	2.94 ± 0.15 ^a^	10 ± 0.50 *	0.67 ± 0.03
11 October	3.43 ± 0.17 ^b^	2.55 ± 0.13 ^b^	5.73 ± 0.29 ^b^	30.4 ± 1.52 *	
12 January	3.32 ± 0.17 ^b^	3.47 ± 0.17 ^c^	9.72 ± 0.49 ^c^	33.17 ± 1.66 ^a^	
12 April	4.29 ± 0.21 ^c^	7.03 ± 0.35 ^d^	8.72 ± 0.44 ^c^	35.03 ± 1.75 ^a^	

All the values are mean ± SD; SD: standard deviation; HE: *n*-Hexan; EA: Ethyl Acetate; ME: methanol; WT: Water (80 °C); CTR: Control (gallic acid). ^a–d^ Column wise values with different superscripts of this type indicate significant difference (*p* <0.05). * EC_40_ value (mg extract mL^−1^ methanol).

The extracts of *Gracilaria* sampled in other months showed higher EC_50_ values than the sample for July (Table 4). In fact, the EC_50_ of the ethyl acetate extract of biomass sampled in October was 2.55 mg mL^−1^, hexane extract was 3.43 mg mL^−1^ and methanol extract was 5.73 mg mL^−1^. The EC_40_ of water extract was 30.4 mg mL^−1^. These values increased in the samples for January and April.

Also the DPPH assay confirmed what already had been observed for the ABTS and FRAP assays. In fact statistical analysis revealed a marked seasonality regarding ethyl acetate extracts. With regard to the *n*-hexane extracts, samples collected in October and January showed comparable values, but significantly different with respect to those collected in the other seasons. Also methanol extracts were characterized by significant differences except for samples collected in spring which were found to be similar to samples collected in winter (Table 4).

Finally, similar to that which we found for ABTS assay, the EC_50_ radical scavenging activity of the ethyl acetate extract of *Gracilaria* sampled in July was very similar to the scavenging activity of BHT used as control (Table 4).

### 2.7. Total Phenolic Content

Phenols are an important group of natural products with antioxidant and other biological activities [57]. These compounds play an important role in algal cell defence against abiotic and biotic stress. Many of them possess antioxidant, antimicrobial and antiviral activities that are important for the protection of algal cells against stress conditions.

Several authors have recently published results regarding the total phenol content and antioxidant activity of algae [51,52].

**Figure 6:**
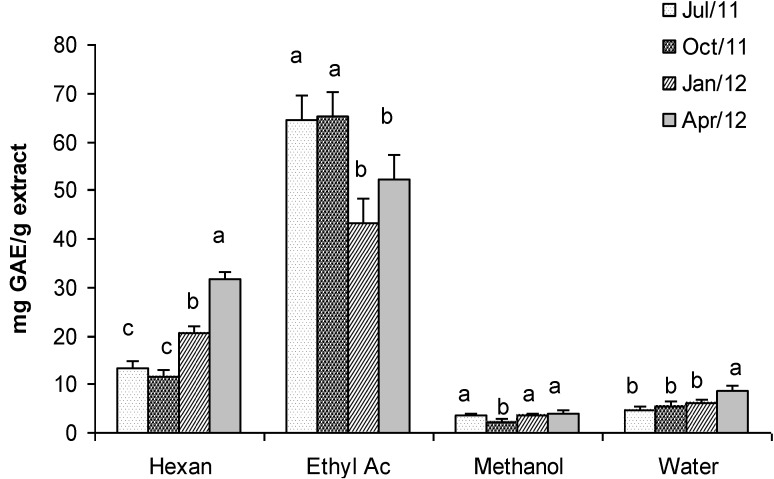
Total Phenolic content (mg GAE g^−1^ dry weight extract) in extracts of *G. gracilis*. Different superscripts (a–d) indicate significant difference (*p* < 0.05).

The phenolic content in extracts of *Gracilaria* was very variable even if a marked seasonality was not so evident, as statistical analyses evidenced (Figure 6). The highest concentration was found in the ethyl acetate extract (65 mg GAE g^−1^ of extract in July and October), whereas the lowest one was found in the methanol extract (2.3 mg GAE g^−1^ of extract in October). Hexane and water extracts showed intermediate values of phenolic content that ranged from 11.56 to 31.8 mg GAE g^−1^ and from 4.7 to 8.8 mg GAE g^−1^ respectively. Interestingly, the phenolic content that we found in ethyl acetate extracts of *Gracilaria gracilis* in July and October was higher than the value reported by Ganesan *et al*. [51] for *Gracilaria edulis* (16.26 mg GAE g^−1^ of extract).

The overlapping of FRAP, TEAC and DPPH assays with the Total Phenols content highlights a positive correlation. In other words, it seems that the antioxidant and scavenging activity is related to phenolic compounds soluble in *n*-hexane and ethyl acetate.

### 2.8. Total Carbohydrates

Carbohydrates perform numerous essential roles in living beings [22]. Monosaccharides are the major source of energy for metabolism while polysaccharides serve for storage of energy and can act as structural components. Furthermore, they have been shown to have other beneficial health effects, including their prebiotic effect and antioxidant or anti-inflammatory activity [58].

**Figure 7:**
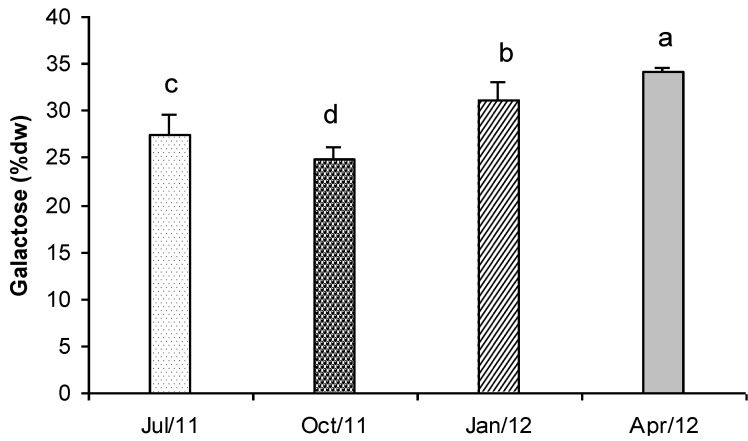
Total carbohydrate content (% dry weight biomass) in *G. gracilis* extract. Different superscripts (a–d) indicate significant difference (*p* < 0.05).

In Figure 7 the concentration of total carbohydrates detected in *Gracilaria* is represented. In April, the average carbohydrates content was 34.1% dry weight, while in October it was 24.8% dry weight. In July and January intermediate concentrations were found (27.5% and 31.1% dry weight respectively). Values showed significant differences in all the tested samples (Figure 7). The seasonal variation and the carbohydrate contents that were found in *Gracilaria* biomass were very similar to those reported by Marinho-Soriano and Bourret [59] for the same species collected in the Thau lagoon (France).

## 3. Experimental Section

### 3.1. Biomass Sampling

*Gracilaria gracilis* was collected from the western area of the Lesina lagoon, where a stable coverage of this seaweed was found (41.866470° N, 15.363350° E). About 3 Kg of wet biomass were sampled in July and October 2011 and January and April 2012, respectively. Algal biomass was washed with distilled water and their epiphytes removed. The fresh seaweed was placed in a freezer (−20 °C) immediately after collection. The cleaned seaweed was freeze-dried at −110 °C for 3 days and then ground to fine powder and stored in airtight containers at −20 °C.

### 3.2. Lipid Extraction

Lipids from the macroalgal pellets were extracted according to Bligh and Dyer [60]. Briefly, 0.5 g of freeze-dried algal biomass were homogenized for 2 min at 12,000 rpm in a mixture of chloroform and methanol (2:1, v/v, 25 mL) using a Kinematica GmgH homogenizer. The mixture was centrifuged and the solid residue re-suspended in a chloroform/methanol mixture (25 mL). Homogenization continued for a further 2 min and the solution was centrifuged again and washed with fresh solvent (25 mL). The combined supernatants were cleaned using a saturated NaCl water solution and the chloroform phase was recovered using a separatory funnel. The water phase was extracted several times with chloroform until the organic solvent was colourless. The chloroform phase was combined and dried with sodium sulphate overnight. The solvent was removed on a rotary evaporator (Büchi Rotavapor) and the purified lipids were weighed.

### 3.3. Saponification of Lipids for Sterols Analysis

Lipids (50 mg under N_2_) were saponified by refluxing in 20 mL of a 5% (w/v) KOH methanol/water (4:1, v/v) solution for 2 h. The refluxed mixture was then transferred into a separatory funnel, and the reflux bottle was washed with 40 mL of Milli-Q water. The unsaponified matter in the combined solution was then extracted four times with 20 mL of *n*-hexane. The hexane phases were then combined, dried with sodium sulphate overnight, filtered and evaporated. Sterols content in the unsaponified fraction was analysed by means of GC-MS.

### 3.4. Sterols Analysis by Gaschromatography-Mass Spectrometry (GC-MS)

Purified sterols fraction of *Gracilaria* biomass was analysed by gas chromatography-mass spectrometry as previously described by Francavilla *et al*. [61]. A Varian Saturn 2200 GC/MS/MS ion trap (Varian Analytical Instruments, Walnut Creek, CA, USA) was used. The GC/MS was equipped with a Varian 3800 CP gas chromatograph (GC). A CP-Sil-8 (30 m × 250 µm × 0.25 µm) fused silica column (Varian Analytical Instruments, Walnut Creek, CA, USA) was installed in the GC and helium was used as carrier gas at a constant flow rate of 1.0 mL min^−1^.

### 3.5. Fatty Acids Methyl Esters (FAMEs) Analysis

Hydroalcolic residue of extraction process of unsaponified fraction was acidified until pH 1 by adding HCl 2 M and extracted 3 times with 25 mL of *n*-hexane. The hexane phases were then combined, dried with sodium sulphate anhydrous, filtered and evaporated. The yellowish viscous liquid obtained (Fatty Acids) was treated with 10 mL of freshly prepared 5% methanolic HCl in a pyrex screw-topped tube. This mixture was carefully mixed and the tube was closed under nitrogen and then heated for 2 h in an oven at 60 °C. After cooling to room temperature, 5 mL of 6% aqueous K_2_CO_3_ were added and the mixture was extracted 4 times with 20 mL of *n*-hexane. The hexane phases were then combined, dried with sodium sulphate anhydrous, filtered and evaporated. The process yielded a yellowish viscous liquid (Fatty Acids Methyl Esters) that was weighed and stored at −20 °C until GC-MS analysis.

FAMEs were analysed by means of the same GC-MS/MS equipment described above for sterols analysis, but with a different column temperature regime: 140 °C for 5 min, followed by a 2 °C/min ramp up to 240 °C, followed by 5 min at 240 °C. The injector temperature was 250 °C and the injected volume was 0.2 µL.

FAME peaks were identified by comparison of their retention times and mass spectra with those of a standard mixture (PUFA C4-C24, Supelco, Bellafonte, PA, USA) whereas they were quantified by using calibration curves made with PUFA C4-C24 standard (Supelco, Bellafonte, PA, USA) and using C15:0 FAME (Supelco, Bellafonte, PA, USA) as internal standard.

### 3.6. Protein Extraction and Analysis

An amount of 50 mg of freeze dried algal sample mixed with 50 mg of alumina was ground manually with pestle and mortar. The mixture was suspended in 5 mL of lysis buffer (0.1 N NaOH, 0.05 M EDTA, 2% SDS, 2% β-mercaptoethanol) [62] for 20 min. After the incubation with buffer, samples were ground for 5 min using a Potter homogeniser (Marconi, model MA099) and kept at room temperature. The mixture was transferred in a centrifuge glass tube and 5 mL of lysis buffer were added to rinse the homogeniser and recover all water-ground material. After this step, samples were centrifuged at 21 °C, 15,000× *g* for 20 min. Supernatants were collected for protein assay. The final volume of the extract was 10.0 mL. The extract was diluted (1:100) in MilliQ water for spectrophotometric analysis according to the Lowry method [63]. The spectrophotometer was blanked with MilliQ water to which was added lysis buffer in the same dilution rate of the samples. Calibration curves were prepared using bovine serum albumin (BSA) (Sigma Co., St. Louis, MO, USA) at the maximum concentration of 100 µg mL^−1^.

### 3.7. Phycobiliprotein Extraction and Analysis

An amount of 0.5 g of freeze dried algal sample mixed with 0.5 g of alumina was ground manually with pestle and mortar. The mixture was suspended in 10 mL of 1 M acetic acid–sodium acetate buffer (pH 5.5) with 0.01% of sodium azide for 30 min in the dark. After the incubation with buffer, samples were ground for 5 min using a Potter homogeniser (Marconi, model MA099). The mixture was transferred to a centrifuge glass tube centrifuged at 5 °C, 15,000× *g* for 20 min. Supernatant was collected and the pellet was extracted three times again with buffer as described. Supernatants were combined and the final volume of the extract was about 40.0 mL. Phycobiliproteins (identified as R-phycoerythrin R-PE, phycocyanin PC and allophycocyanin APC) were quantified by spectrophotometry according to Kursar *et al*. [64].

The absorbance of the aqueous supernatants was determined at 498.5, 614.0, and 651.0 nm and their APC, PC, and R-PE contents were calculated as µg mL^−1^ using the following Equations (1):

APC = 181.3*A*_651_ − 22.3*A*_614_
PC = 151.1*A*_614_ − 99.1*A*_651_
R-PE = 155.8*A*_498.5_ − 40.0*A*_614_ − 10.5*A*_651_ (1)

### 3.8. Fractionated Extraction of Algal Biomass

The extraction procedure of Hajimahmoodi *et al*. [65] was performed. Four solvents with different polarity were used to perform the extractions. They were conveniently selected in order to perform selective extraction of compounds (or better, group of compounds) with different polarities, according to the principle “*similia solunt similibus*”. Briefly, a precisely weighed amount (2 g) of ground dried algal biomass was homogenized at 17,000 rpm, using a Kinematica GmgH homogenizer, twice each time with 20 mL of *n*-hexane at room temperature for 1 min followed by centrifugation. Further extractions in a similar manner were performed sequentially on the pellet using ethyl acetate, methanol and then deionized water (at 80 °C). The isolated supernatants for each solvent were combined. Hexane, ethyl acetate and methanol extracts were evaporated under vacuum and the dried samples were weighed, dissolved in 1 mL of methanol and stored at −20 °C until analysis. The water extract was freeze dried, weighed and dissolved in 5 mL of methanol. The four extracts were used for antioxidant activity assay and analysis of the total phenolic content.

### 3.9. Antioxidant Activity Assays

#### 3.9.1. Reducing Power: The FRAP Assay

The FRAP (ferric-reducing antioxidant power) assay is based on the reduction of a ferric-tripyridyl triazine complex to its ferrous-blue-coloured form in the presence of antioxidants [66]. Briefly, the FRAP reagent contained 5 mL of a TPTZ (2,4,6-tripyridyl-*S*-triazine) solution (10 mmol L^−1^) in HCl (40 mmol L^−1^) plus 5 mL of FeCl_3_ (20 mmol L^−1^) and 50 mL of acetate buffer (0.3 mol L^−1^, pH 3.6). It was freshly prepared and warmed to 37 °C. A 100 μL sample of each extract (hexane, ethyl acetate, methanol and water) was mixed with 3 mL of FRAP reagent and the absorbance of the reaction mixture was measured at 593 nm after incubation at 37 °C for 10 min. The results could be expressed in micromole of Fe (II), (+) catechin, vitamin C, Trolox or BHT equivalent [67]. For the present study, the standard curve was constructed using Trolox solution (1–30 µM) and the results were expressed as µmol Trolox g^−1^ dry weight of extract. Butylated hydroxytoluene (BHT) whose concentration was 0.1 mg mL^−1^ was used as positive control.

#### 3.9.2. ABTS Assay

The antioxidant activities of seaweed extracts were also measured by using the ABTS [2-2′-azino-bis (3-ethylbenz-thiazoline-6-sulfonic acid)] assay as described by Matanjun *et al*. [52]. ABTS is a chromogen (colourless) that would be converted to blue-green coloured ABTS^+^ radical cation by an oxidative reagent. ABTS^+^ could also be reduced to its colourless form by antioxidant. The absorbance was measured spectrophotometrically at 645 nm as a function of concentration and the scavenging percentage of ABTS^+^ was calculated relative to Trolox, a water-soluble analogue of vitamin E used as an antioxidant standard.

Briefly, ABTS^+^ radical cation was generated by a reaction of 7 mM sample with 2.45 mM potassium persulphate. The reaction mixture was allowed to stand in the dark for 16 h at room temperature. A working solution was prepared by diluting 3 mL of ABTS stock solution with 57 mL phosphate buffered saline (PBS, 5 mM, pH 7.4). A volume of 2 mL of this working solution was dispensed to test tubes. Addition of 200 µL diluted methanolic extracts (10 µL of extract was diluted with 190 µL of methanol, dilution 1:20) initiated the reaction and absorbance was read after exactly 6 min. Gallic acid (GA) at the concentration of 0.1 mg mL^−1^ was used as positive control. The results were expressed as mmol Trolox g^−1^ dry weight of extract.

#### 3.9.3. DPPH Radical Scavenging Activity

This activity was detected by the method of Hu *et al*. [68]. An aliquot of each sample (30 µL, 0.5–30 mg/mL) in acetone/MeOH (1/1, v/v) was mixed with 200 µL of 100 µM DPPH (2,2-diphenyl-2-picrylhydrazyl hydrate) prepared with methanol. The mixture was shaken vigorously and then left to stand at room temperature for 60 min in the dark. The absorbance was measured spectrophotometrically at 520 nm against an acetone/MeOH (1/1, v/v) blank. The lower absorbance indicated the stronger scavenging activity. EC_50_ value (mg extract mL^−1^), the effective concentration at which 50% of the DPPH radicals were scavenged, was obtained from the plot of scavenging activity against the concentration of extract. The scavenging activity was calculated based on the percentage of DPPH radical scavenged using the follow Equation (2) [50]:

Scavenging effect (%) = [1 − (*A*_sample_ − *A*_sample blank_)/*A*_control_] × 100 (2)

where the *A*_control_ is the absorbance of the control (DPPH solution without extract), the *A*_sample_ is the absorbance of the test extract (DPPH solution plus test extract), and the *A*_sample blank_ is the absorbance of the extract only (extract without DPPH solution). Butylated hydroxytoluene (BHT), a synthetic antioxidant, was used as positive control.

### 3.10. Determination of Total Phenolic Content

Total phenolics were colorimetrically determined using Folin-Ciocalteu reagent as described by Velioglu *et al*. [69] with slight modifications. The extract (200 µL) was mixed with 1.5 mL of Folin-Ciocalteu reagent (previously diluted tenfold with distilled water) and allowed to stand at room temperature for 5 min. A 1.5 mL sodium bicarbonate solution (60 g L^−1^) was added to the mixture. After incubation for 90 min at room temperature, the absorbance was measured at 750 nm. Total phenolics were quantified by a calibration curve obtained from measuring the absorbance of known concentrations of gallic acid standard solutions (25–150 µg mL^−1^ in 50% methanol). The results were calculated as gallic acid equivalent (GAE) g^−1^ dry weight of extract.

### 3.11. Total Carbohydrates

Total carbohydrate content was assayed by the phenolsulphuric acid method [70] after extraction with 2.5 N HCl for 3 h at 100 °C. The results were calculated from a galactose standard curve and reported as % dry weight of algal biomass.

### 3.12. Statistical Analyses

All the experiments were repeated three times. Unless otherwise stated, all data were expressed as mean ± standard deviation (SD). The means of all the parameters were examined for significance by analysis of variance (ANOVA) using the software JMP version 9 (SAS Institute Inc., Cary, NC, USA). When *F* values showed significance, individual means were compared using Tukey’s honest significant difference (HSD). Significant differences were considered when *p* < 0.05.

## 4. Conclusions

*Gracilaria gracilis* of Lesina Lagoon is a natural marine biomass which has already been found interesting for several applications including agar, as mesoporous material and for bio-oils production. The results of this study highlight that this marine biomass seems to be also a promising source of R-phycoerythrin if it is harvested in winter time. In the same season, the red seaweed also contains a high amount of other valuable products including arachidonic acid (PUFA ω-6), proteins and carbohydrates. High antioxidant and radical scavenging activity, comparable to that of commercial antioxidants compounds, was found in biomass harvested in summer time when the highest concentration of total phenols was also detected. However, more investigations are required for separating, purifying and characterizing these bioactive compounds. Furthermore, it will be necessary to deepen the knowledge of the biology of this macroalga in the Lesina Lagoon, by considering the life cycle and the reproductive peaks to optimize the exploitation of the species and to safeguard its presence in the lagoon.

In conclusion, this study pointed out the possibility of using *Gracilaria gracilis* as a *multi products source* for biotechnological, nutraceutical and pharmaceutical applications according to the new concept of “Biorefinery”. Nevertheless, it will be very important to investigate the economic and environmental affordability and sustainability of *Gracilaria* natural resource by means of Life Cycle Assessment.
